# Improving Tibial Component Coronal Alignment During Total Knee Arthroplasty with the Use of a Double‐Check Technique

**DOI:** 10.1111/os.12570

**Published:** 2019-11-22

**Authors:** De‐si Ma, Zhi‐wei Wang, Liang Wen, Shi‐xiang Ren, Yuan Lin, Bo Zhang

**Affiliations:** ^1^ Department of Orthopedics, Beijing Chaoyang Hospital Capital Medical University Beijing China

**Keywords:** Knee, Arthroplasty, Osteotomy, Malalignment, Tibia

## Abstract

**Objective:**

To compare the efficacy of the restoration of tibial component coronal alignment with a double‐check technique and the conventional surgical technique during total knee arthroplasty (TKA) in knee osteoarthritis patients, and to investigate the distribution of the medial proximal tibial angle (MPTA) after TKA.

**Methods:**

A retrospective review was performed of 151 patients (179 knees) with knee osteoarthritis undergoing primary TKA in Beijing Chaoyang Hospital, Capital Medical University from February 2013 to January 2015 to evaluate the differences in MPTA in patients undergoing the conventional TKA and those undergoing a modified TKA with a double‐check technique after the surgery. All patients were evaluated by MPTA, range of motion (ROM), Knee Society Clinical Rating System (KSS) clinical scores, and KSS functional scores. An MPTA deviation of 3° or greater was considered malalignment.

**Results:**

A total of 130 TKA procedures in 119 patients were included in the study: 64 knees treated with conventional TKA and 66 knees treated with the double‐check technique TKA. The mean postoperative MPTA was 88.6° ± 2.2° in the conventional TKA group and 89.1° ± 1.5° in the double‐check TKA group. The mean postoperative MPTA between the two groups was not significantly different. In the conventional TKA group, 79.7% (51 knees) had a postoperative MPTA deviation within 3° and 20.3% (13 knees) had a MPTA deviation greater than 3°. In the double‐check TKA group, 93.9% (62 knees) had a postoperative MPTA deviation within 3°and 6.1% (4 knees) had a MPTA deviation greater than 3°. The postoperative MPTA deviation within 3° showed a statistically significant difference between the two groups. In the double‐check TKA group, a 21.2% (14 knees) tibial malalignment was detected after the first check and a 9.1% (6 knees) tibial malalignment was detected after the second check. The mean postoperative ROM was 118.1° ± 9.2° in the conventional TKA group and 115.7° ± 10.1° in the double‐check TKA group. The mean postoperative KSS clinical score was 89.3 ± 3.5 in the conventional TKA group and 89.0 ± 3.7 in the double‐check TKA group. The mean postoperative KSS functional score was 84.8 ± 10.0 in the conventional TKA group and 84.9 ± 9.0 in the double‐check TKA group. The mean postoperative ROM, KSS clinical scores, and KSS functional scores between the two groups were not statistically significantly different.

**Conclusion:**

Malalignment of the tibial component can occur after conventional TKA, and the double‐check technique is an effective method to improve tibial component coronal alignment.

## Introduction

Total knee arthroplasty (TKA) has been one of most successful surgeries over the past several decades. TKA can help patients to regain knee function, enhance the muscle force of lower limbs, relieve the pain of knees and improve patients’ general quality of life. It is acknowledged that implant alignment is one of the key factors in the outcome of TKA. Therefore, the restoration of a neutral mechanical axis of the lower limbs is among the most important objectives of TKA during surgery procedures. Several studies have demonstrated that implant malalignment impacts bone and implant stress, polyethylene wear, knee function, and long‐term implant survival, and even leads to early failure of TKA. Most surgeons consider that implant alignment parallel to the mechanical axis within 3° deviation shows better performance than alignment outliers^1^, ^2^, ^3^, ^4^, ^5^. In last ten years, a series of improvements for surgical instrumentation and implant design have been developed to achieve the ideal TKA alignment. However, the outcome is not completely satisfactory. Compared with the complex femoral axis, it is easier to improve the tibial alignment. However, malalignment of the tibial component is not uncommon, and even reaches 35% in some cases^6^, ^7^. Intramedullary and extramedullary alignment are the general techniques of TKA. Whether the intramedullary alignment is superior to extramedullary alignment remains controversial. Another important question is whether intramedullary alignment can be performed if a severe malformation has occurred on the tibial shaft. As a result, most surgeons like to use the extramedullary technique as a routine method to undertake the tibial alignment. To achieve accurate alignment and ideal outcomes for TKA, some new techniques have been developed. Computer‐assisted surgical navigation is designed to help surgeons obtain accurate mechanical alignment using sensors to confirm the position of the mechanical axis during surgeries and its application is becoming increasingly popular. Some studies have shown that it can achieve better function, faster rehabilitation, and improved quality of life, whereas others have not demonstrated any clinical or functional benefits. At the same time, computer‐assisted surgical navigation could lead to new problems, such as higher cost, longer surgery time, and more complexity^5^, ^8^, ^9^, ^10^, ^11^. Patient‐specific instrumentation could reduce many steps during TKA and aims to improve mechanical alignment through preoperative planning and 3‐D printing techniques. In addition, there are no advantages in restoration of the mechanical axis versus conventional instrumentation on the tibial side^12^. Moreover, patient‐specific instrumentation requires extra CT scans or MRI, and 3‐D printing equipment. Therefore, it cannot be widely used.

The tibial component alignment includes the coronal plane, the sagittal plane, and the rotation of the cross‐section. Each of the three is critical for the outcome. Compared with the other two, the alignment in the coronal plane has the exact landmark that could be confirmed on the radiograph and the measurement is repeatable. The sagittal plane alignment is not easy to measure precisely on the radiographs after surgery. Besides, it is impossible to measure the rotation of the tibial component in the cross‐section using X‐ray radiographs. It has been reported that preparation of the tibia during TKA may cause deviation from the tibial mechanical axis and lead to tibial component malalignment in the coronal plane. Methods to confirm the tibial mechanical axis^13^, inaccurate bone cuts^14^, ^15^, and attempts to implant the tibial implant are the potential causes of malalignment^16^, ^17^. After the osteotomy and soft tissue balancing, femoral and tibial trials could be used to examine the axis; this is typically the last opportunity to confirm the alignment of the tibial component during conventional TKA procedures. Nevertheless, when the component is implanted, different thicknesses of cement and trabecular collapse caused by hammering still present a potential risk for malalignment. Conventional TKA procedures cannot detect the malalignment after the trials have been tested. In addition, if malalignment occurs, no adjustment can be performed when the tibial component is implanted.

To improve tibial component alignment in the coronal plane, we used a double‐check technique with a simple device to detect the malalignment and perform adjustments during conventional TKA procedures. Therefore, the purpose of the present study was as follows: (i) to compare the clinical efficacy for tibial component alignment in the coronal plane by conventional TKA and the double‐check technique TKA in osteoarthritis patients; (ii) to investigate the distribution of tibial component alignment in the coronal plane after conventional TKA and double‐check technique TKA; and (iii) to discuss the clinical outcomes and advantages of the application of the double‐check technique TKA.

## Materials and Methods

### *Inclusion and Exclusion Criteria*

#### *Inclusion Criteria*

Inclusion criteria were: (i) patients with knee osteoarthritis; (ii) patients who had undergone conventional TKA or the double‐check TKA; (iii) patients with adequate radiographs to measure the alignment of lower limbs; and (iv) a retrospective case control study.

#### *Exclusion Criteria*

Exclusion criteria were: (i) history of fractures around the knee or femoral and tibial shaft; (ii) patients with valgus deformities of the knee; (iii) patients with poor‐quality radiographs; (iv) patients who underwent revision surgeries after primary TKA; and (v) patients lost to follow‐up.

### *General Information of Participants*

A retrospective review was performed of 151 patients (179 knees) with osteoarthritis undergoing primary TKA by one single surgeon in our institution from February 2013 to January 2015. A cruciate retaining prosthesis with mobile bearing design (Gemini Mark II, LINK, Germany) was implanted in all patients using a cementing technique. All patients were evaluated preoperatively and 3 months postoperatively with an anteroposterior long‐leg weight‐bearing radiograph. The medial proximal tibial angle (MPTA) was measured by radiograph.

### *Surgical Technique*

#### *Conventional Total Knee Arthroplasty Procedure*

The standard TKA procedure with conventional instrumentation was performed in the conventional TKA group. An anterior midline skin incision and a medial parapatellar approach was used, and the patella was everted. A measured resection technique was used to balance the extension and flexion gap. A distal femoral cut was performed with an intramedullary instrumentation setting of 6° of the anatomic valgus. Referring to the surgical trans‐epicondylar axis, the femoral external rotation cut was performed. Extramedullary instrumentation was used to achieve a target tibial cut of 90° relative to the mechanical axis in the coronal plane and of 3° to 5° relative to the posterior slope in the sagittal plane. After appropriate soft‐tissue release, trials of the tibial and femoral side were installed to test stability, range of motion, patellar tracking, and alignment of the lower limbs. Next, the prosthesis was fixed with cement. No patellar resurfacing was performed in any of our cases.

#### *Total Knee Arthroplasty with Double‐Check Technique*

**Figure 1:**
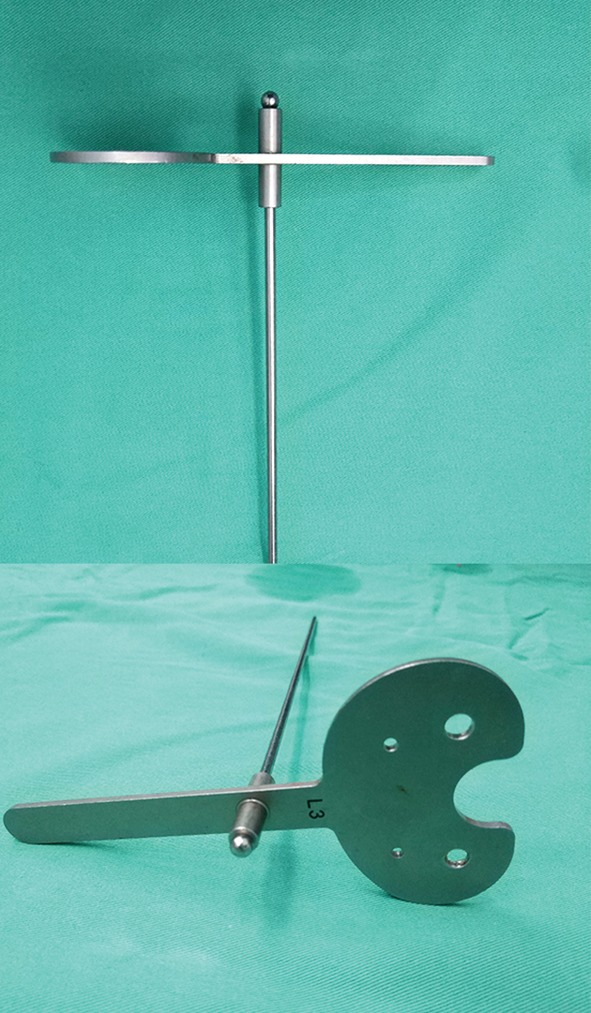
The device used in the double‐check technique, including a smooth surface plate and an extramedullary rod.

**Figure 2:**
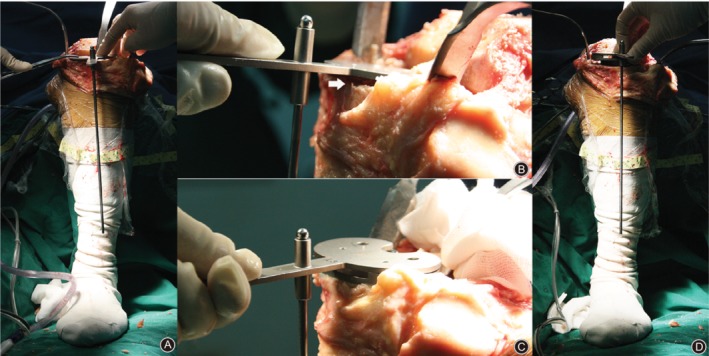
Intraoperative photographs illustrating the steps of the double‐check technique. (A) The extramedullary rod is not parallel to the mechanical axis of the tibia in the coronal plane when the first check is performed. (B) The gap between the device and bone surface. (C) The second check when the tibial tray is implanted with doughy cement. (D) The tibial component is now correctly aligned.

In the double‐check TKA group, most of the steps were the same as those in the conventional TKA group, except for the double‐check technique. The first check was performed after the tibial cut was finished. A device with a smooth surface plate was used to evaluate the flatness of the bone surface (Fig. 1). If the extramedullary rod was not parallel to the mechanical axis of the tibia in the coronal plane, an uneven bone surface was confirmed. Moreover, a gap could be observed between the device and bone surface (Fig. 2). Then, a re‐cut was performed until the bone surface was smooth and accurate alignment was obtained. The second check was performed when the tibial tray was implanted with cement (Fig. 2); different thicknesses of cement, trabecular collapse, and the subsidence of the tibial tray caused by hammering could affect the accuracy of alignment. If malalignment occurred, a suitable adjustment could be performed by hammering on the proper position of the tibial tray.

### *Measurements*

#### *Definition of Medial Proximal Tibial Angle*

**Figure 3:**
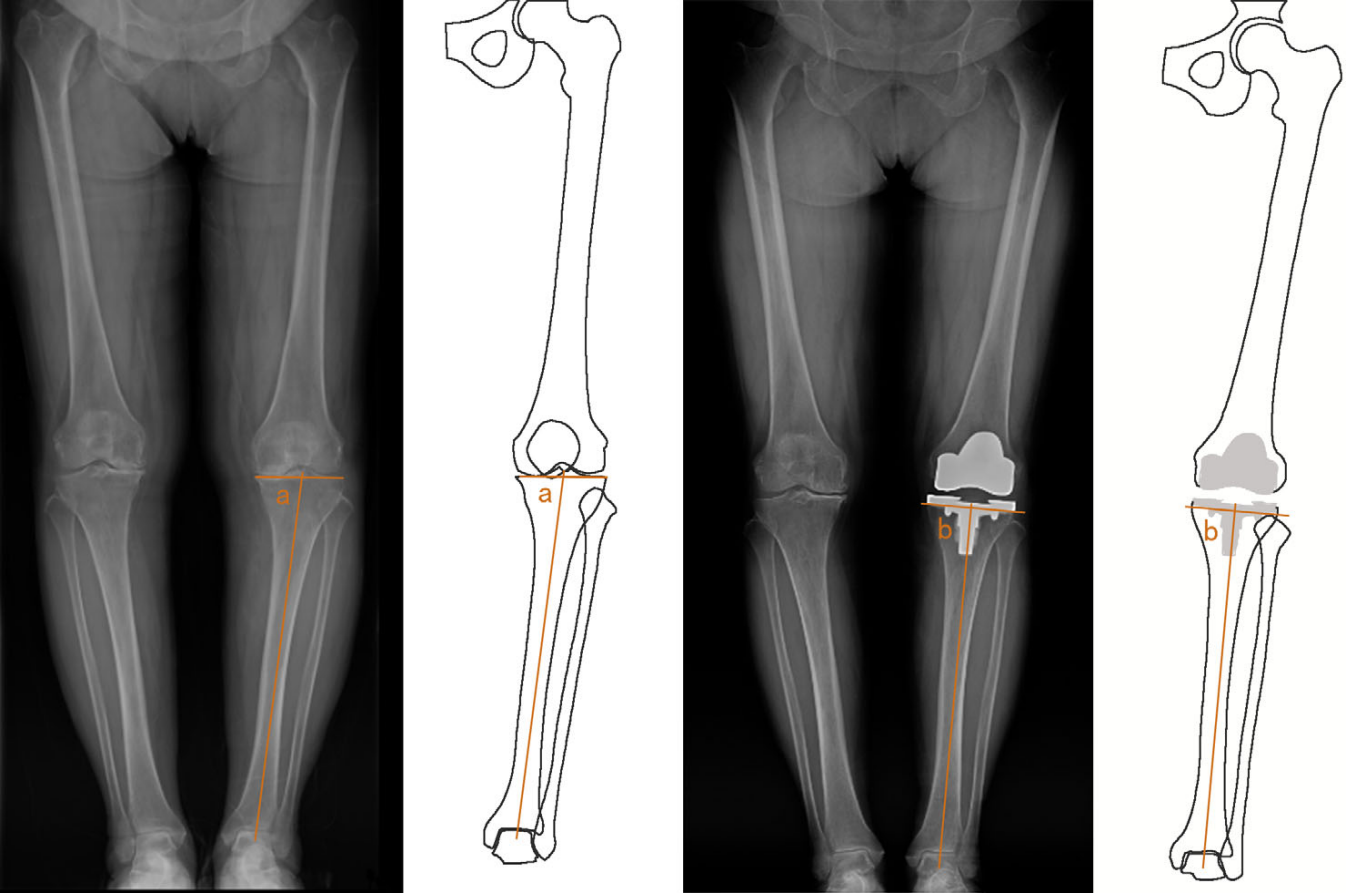
Radiograph showing the measurement of MPTA. MPTA is the medial angle formed by the tibial mechanical axis and frontal plane joint line of the proximal tibia (MPTA before and after surgery).

On the anteroposterior long‐leg weight‐bearing radiographs, the tibial mechanical axis was defined by a line joining the center of the proximal tibia and the center of the body of the talus. The frontal plane joint line of the proximal tibia was drawn preoperatively across the flat or concave aspect of the subchondral line of the two tibial plateaus. The frontal plane joint of the proximal tibia was drawn parallel to the undersurface of the tibial component postoperatively. The MPTA was the medial angle formed by these two lines^18^. The varus alignment was classified as MPTA less than 90°, and the valgus alignment was classified as MPTA greater than 90° (Fig. 3).

#### *Measurements of Medial Proximal Tibial Angle*

Measurements were performed by two doctors separately. The mean of the two measurements was recorded for each radiograph. If the disagreement was greater than 1°, the two measurements would repeat until the disagreement was 1° or less.

#### *Knee Society Clinical Rating System Scores*

The Knee Society Clinical Rating system (KSS) included clinical scores (pain and stability) and functional scores (walking and climbing stairs) for the knee. KSS scores were evaluated at the time of latest follow‐up.

#### *Knee Range of Motion*

Knee range of motion (ROM) was measured using a goniometer. Patients lay down on the back on a firm surface. Knee lateral epicondyle, lateral malleolus, and great trochanter were the landmarks to do the angle measurements. ROM was recorded at the time of latest follow up.

## Statistical Analysis

To determine the minimum sample size of each group, a power analysis was conducted before the beginning of the study. The minimum sample size of each group was 62 knees. (α = 0.05, β = 0.10)^19^. The measurement data, including age, follow‐up time, MPTA, ROM, KSS clinical scores, and KSS functional scores were represented by mean ± standard deviation, and were statistically analyzed using *t*‐tests. The distribution of MPTA was statistically analyzed using *χ* ^2^‐tests as the categorical data. Statistical Package for the Social Sciences (SPSS, IBM, USA) version 19.0 was used for statistical analysis. The level of significance was set at *P* ≤ 0.05.

## Results

### *Patients Characteristics*

A total of 130 TKA procedures in 119 patients were included in the study. These patients included 35 men and 84 women and the mean age was 71.5 ± 5.3 years old. The mean follow‐up time was 36.2 ± 5.8 months (13–55 months). The characteristics for the two groups are shown in Table 1.

**Table 1:** Patients’ characteristics according to treatment group

Variable	Conventional TKA group	Double‐check TKA group	*P*‐value
Number of knees/patients	64/60	66/59	‐
Sex (male/female)	19/41	16/43	‐
Age (year)	71.3 ± 6.2	71.5 ± 5.9	0.93
Follow‐up (months)	35.2 ± 6.0	37.2 ± 5.4	0.79

The data are presented as mean ± standard deviation. TKA, total knee arthroplasty.

### *Knee Range of Motion*

There was no significant difference in the mean value of preoperative ROM between the two groups. In addition, there was also no significant difference in the mean value of postoperative ROM. The ROM was significantly improved after surgeries in both group (12.5% in the conventional TKA group and 13.0% in the double‐check TKA group). The results are shown in Table 2.

**Table 2:** Knee ROM according to treatment group (°)

Group	Before surgery	After surgery	*P*‐value
Conventional TKA	104.9 ± 16.0	118.1 ± 9.2	0.00
Double‐check TKA	102.4 ± 17.1	115.7 ± 10.1	0.00
*P*‐value	0.40	0.8	—

The data are presented as mean ± standard deviation. ROM, range of motion; TKA, total knee arthroplasty.

### *Knee Society Clinical Rating System Scores*

There were no significant differences in the mean values of preoperative KSS clinical scores and KSS functional scores between the two groups. There were also no significant differences in the mean values of postoperative KSS clinical scores and KSS functional scores. The KSS clinical scores and KSS functional scores were significantly improved after surgeries in both groups (90.4% and 85.6%, respectively, in the conventional TKA group and 85.0% and 83.4%, respectively, in the double‐check TKA group). The results are shown in Table 3.

**Table 3:** KSS scores according to treatment group

	KSS clinical scores			KSS functional score		
Group	Before surgery	After surgery	*P*‐value	Before surgery	After surgery	*P*‐value
Conventional TKA	46.9 ± 9.3	89.3 ± 3.5	0.00	45.7 ± 9.9	84.8 ± 10.0	0.00
Double‐check TKA	48.1 ± 9.0	89.0 ± 3.7	0.00	46.3 ± 14.1	84.9 ± 9.0	0.00
*P*‐value	0.23	0.57	‐	0.91	0.17	‐

The data are presented as mean ± standard deviation. KSS, The Knee Society Clinical Rating system.

### *Comparison of Medial Proximal Tibial Angle*

There were no significant differences in the mean values of preoperative MPTA, between the two groups. There were also no significant differences between the mean values of postoperative MPTA. The MPTA was significantly improved after surgeries in both group (Table 4).

**Table 4:** MPTA according to treatment group (°)

Group	Before surgery	After surgery	*P*‐value
Conventional TKA	84.5 ± 3.6	88.6 ± 2.2	0.00
Double‐check TKA	85.1 ± 3.9	89.1 ± 1.5	0.00
*P*‐value	0.40	0.17	‐

The data are presented as mean ± standard deviation. MPTA, medial proximal tibial angle; TKA, total knee arthroplasty.

**Figure 4:**
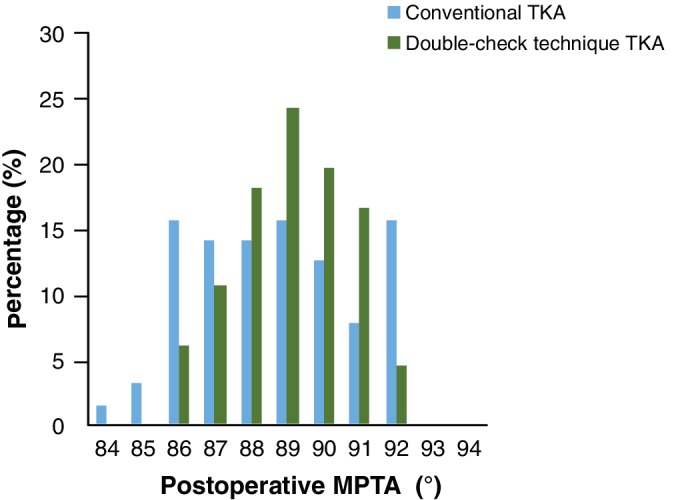
The distribution of the postoperative medial proximal tibial angle (MPTA) for the conventional total knee arthroplasty (TKA) group and the double‐check TKA group.

In the conventional TKA group, 79.7% (51 knees) of postoperative MPTA deviation was within 3°, and 20.3% (13 knees) had a deviation greater than 3°. In the double‐check TKA group, 93.9% (62 knees) of postoperative MPTA deviation was within 3°, and 6.1% (4 knees) had a deviation greater than 3°. There was a statistically significant difference between the two groups (*χ*2 = 5.86, *P* < 0.05). The accuracy of postoperative MPTA in double‐check TKA was 14.2% higher than in the conventional group. The distribution of postoperative MPTA for the two groups is shown in Fig. 4.

### *Outcome of Double Check*

In the double‐check TKA group, a 21.2% (14 knees) tibial malalignment was detected after the first check, and the adjustment was performed. Tibial component malalignment was detected after the second check in 9.1% (6 knees), and the adjustment was performed. In the double‐check TKA group, 4.5% (3 knees) undergoing TKA required double adjustment after the first and second checks were performed. Among the four outliers in the double‐check TKA group, two knees were adjusted after the first check and no malalignment was detected after the double‐check in the remaining two knees.

## Discussion

### *Improvement of the Tibial Component Coronal Alignment*

The most important finding of this study is that, in performing a TKA, the coronal alignment of the tibial component could be improved using a surgical technique in which the double‐check technique is performed after the tibial cut and tibial tray cementing. Although the mean value of postoperative MPTA was similar between the group that was treated with conventional instrumentation alone and the group that was treated with the use of the double‐check technique, the deviation within 3° from the target coronal alignment was obviously reduced in the double‐check TKA group, as evidenced by the reduced variation in data.

### *Causes of Malalignment*

Proximal tibial resection is a vital step during TKA, and initial bone cutting error of the proximal tibia has already been investigated. Toksvig‐Larsen^20^ reported a maximum roughness between the uppermost and lowermost points of 1.0 mm and 2.4 mm, respectively, for the bone surface on a cadaveric tibial plateau. The bending and wobbling of the saw, areas of sclerotic bone, unstable cutting blocks, and imprecise surgical technique might introduce errors in the tibial cut^14^, ^19^, ^21^. In the present study, the same conclusion was drawn. Tibial malalignment was detected after the tibial osteotomy in 21.2% (14 knees) and in 9.1% (6 knees) of cases after the second check, when the tibial tray was implanting with cement. We inferred from this result that different thicknesses of cement trabecular collapse and the subsidence of the tibial tray caused by hammering when implanting the tibial component could lead to the tibial malalignment. According to the trigonometric function, a 0.5‐mm protuberance in the middle of a 70‐mm length of tibial cutting surface could cause a 0.82° error. In addition, if the protuberance is 1 mm, the error will be 1.6°, which may cause the failure of tibial alignment in the coronal plane.

Previous studies have confirmed that these errors could lead to the tibial malalignment, and several techniques have been used to solve this problem. A prospective randomized, controlled trial compared the cutting accuracy of a precision saw system with the conventional blade system during TKA. The result was that the precision saw system was not proven to be overall more accurate than the conventional blade system. In addition, surgeon factors seemed not to have a major role in the accuracy of the bone resection^14^. Macdonald et al^21^ reported that modified sawblades (thicker and stiffer), a new saw guide, and the technique of constraining the saw from the pivot point of the blade improved the result of tibial cutting compared with the conventional sawing techniques. Wu et al^22^ reported that preoperative measurement of the difference between the resection thicknesses of the medial and lateral tibial plateaus for the proximal tibial cut could improve the accuracy of tibial component alignment and postoperative limb alignment restoration compared with conventional techniques. Cinotti et al^13^ reported that the alignment of the tibial component may be improved using a surgical technique in which the extramedullary rod is set in line with anatomical landmarks in the proximal tibia only without any other instrumentation.

### *Advantages of the Double‐Check Technique*

The reasons for the inaccuracy of bone cuts and malalignments are complicated. A block with an extramedullary rod to insert the extension gap is used to check the coronal alignment for most of the instrumentation. The femoral resection could influence the result. At the same time, it is difficult to confirm the center of the femoral head during the surgery. The present study focuses on the tibial coronal alignment, regardless of the effect of the femoral side. The second check can't be performed for most of the prosthesis after the tibial component is implanted. Different thicknesses of cement, trabecular collapse, and the subsidence of the tibial tray caused by hammering could affect the accuracy of alignment. In other words, although an accurate tibial cut is performed, a fatal MPTA could be obtained during the implanting procedure. Tibial tray malalignment was detected after the second check in our study, and this is the first report about it as far as we know. The double‐check technique was used to evaluate the MPTA after the two important steps, regardless of the cause of malalignment, and the outcome was encouraging in the present study.

Computer‐assisted surgical navigation and patient‐specific instrumentation in TKA are the current treatment trends. They help surgeons achieve accurate TKA and the ideal alignment of the lower limbs. However, the results are controversial^5^, ^8^, ^9^, ^10^, ^11^, ^12^, ^23^, ^24^. The advantages of computer‐assisted surgical navigation and patient‐specific instrumentation are to identify the mechanical axis during the surgery based on the preoperative planning. They can reduce the human error to confirm the mechanical axis effectively. At the same time, they involve new systematic errors caused by the additional instrumentation. Meanwhile, the bone cutting deviations and errors caused by cementing are inevitable. Because of longer surgery time, increased cost, and highly sensitive instruments, computer‐assisted surgical navigation and patient‐specific instrumentation in TKA are not the first choice for most surgeons, especially in developing countries.

### *Medial Proximal Tibial Angle Distribution*

If we look at the distribution of postoperative MPTA more precisely (Fig. 3), no matter whether in the conventional TKA group or the double‐check TKA group, the distribution trend is slightly greater for varus than valgus. Other reports show the same situation^13^, ^19^, ^22^. It has been reported that residual varus alignment after TKA in varus knees is acceptable, but valgus alignment after TKA in varus knees is not allowed^25^, ^26^. To avoid unacceptable valgus malalignment after TKA in varus knees, surgeons may potentially restore a little bit of varus alignment as they see fit.

### *Limitations*

There are several limitations in this study. First, it is a retrospective, nonrandomized, single‐center study, with all the limitations of such a study design. Second, sagittal alignment and rotation of the tibial component are not included in this study, which are very important for knee function after TKA^13^, ^27^. Finally, although the double‐check technique TKA could improve the coronal alignment of the tibial component compared to the conventional TKA, there was no significant difference in the clinical outcomes (ROM, KSS clinical scores, and KSS functional scores) between the two groups during the follow up. To evaluate whether the survival rate and clinical outcomes are associated with MPTA after TKA, a long‐term follow‐up study needs be conducted.

### *Conclusion*

In conclusion, these results provide evidence for the efficacy of the double‐check technique, which could improve tibial component coronal alignment during TKA. There are no disadvantages (e.g. in relation to expense and complexity, or the need for additional CT scans or MRI) of computer‐assisted surgical navigation and patient‐specific instrumentation. This technique could be recommended for routine use in TKA.

## References

[1] Innocenti B , Bellemans J , Catani F . Deviations from optimal alignment in TKA: is there a biomechanical difference between femoral or tibial component alignment? J Arthroplasty, 2016, 31: 295–301. 26321075 10.1016/j.arth.2015.07.038

[2] Berend ME , Ritter MA , Meding JB , *et al* Tibial component failure mechanisms in total knee arthroplasty. Clin Orthop Relat Res, 2004, 428: 26–34. 10.1097/01.blo.0000148578.22729.0e 15534515

[3] Collier MB , Engh CA Jr , McAuley JP , Engh GA . Factors associated with the loss of thickness of polyethylene tibial bearings after knee arthroplasty. J Bone Joint Surg Am, 2007, 89: 1306–1314. 17545435 10.2106/JBJS.F.00667

[4] Ritter MA , Faris PM , Keating EM , Meding JB . Postoperative alignment of total knee replacement. Its effect on survival. Clin Orthop Relat Res, 1994, 299: 153–156. 8119010

[5] Choong PF , Dowsey MM , Stoney JD . Does accurate anatomical alignment result in better function and quality of life? Comparing conventional and computer‐assisted total knee arthroplasty. J Arthroplasty, 2009, 24: 560–569. 18534397 10.1016/j.arth.2008.02.018

[6] Reed MR , Bliss W , Sher JL , Emmerson KP , Jones SM , Partington PF . Extramedullary or intramedullary tibial alignment guides: a randomised, prospective trial of radiological alignment. J Bone Joint Surg Br., 2002, 84: 858–860. 12211678 10.1302/0301-620x.84b6.12702

[7] Teter KE , Bregman D , Colwell CW Jr . Accuracy of intramedullary versus extramedullary tibial alignment cutting systems in total knee arthroplasty. Clin Orthop Relat Res, 1995, 321: 106–110. 7497654

[8] Kim YH , Kim JS , Choi Y , Kwon OR . Computer‐assisted surgical navigation does not improve the alignment and orientation of the components in total knee arthroplasty. J Bone Joint Surg Am, 2009, 91: 14–19. 19122074 10.2106/JBJS.G.01700

[9] Macule‐Beneyto F , Hernandez‐Vaquero D , Segur‐Vilalta JM , *et al* Navigation in total knee arthroplasty. A multicenter study. Int Orthop, 2006, 30: 536–540. 16736149 10.1007/s00264-006-0126-7 PMC3172750

[10] Barrett WP , Mason JB , Moskal JT , Dalury DF , Oliashirazi A , Fisher DA . Comparison of radiographic alignment of imageless computer‐assisted surgery vs conventional instrumentation in primary total knee arthroplasty. J Arthroplasty., 2011, 26: 1273–1284. 21723703 10.1016/j.arth.2011.04.037

[11] Todesca A , Garro L , Penna M , Bejui‐Hugues J . Conventional versus computer‐navigated TKA: a prospective randomized study. Knee Surg Sports Traumatol Arthrosc, 2017, 25: 1778–1783. 27306985 10.1007/s00167-016-4196-9

[12] Sharareh B , Schwarzkopf R . Review article: patient‐specific versus standard instrumentation for total knee arthroplasty. J Orthop Surg (Hong Kong), 2015, 23: 100–106. 25920655 10.1177/230949901502300123

[13] Cinotti G , Sessa P , D'Arino A , Ripani FR , Giannicola G . Improving tibial component alignment in total knee arthroplasty. Knee Surg Sports Traumatol Arthrosc, 2015, 23: 3563–3570. 25218573 10.1007/s00167-014-3236-6

[14] Feczko PZ , Fokkenrood H , van Assen T , Deckers P , Emans PJ , Arts JJ . Accuracy of the Precision Saw versus the Sagittal Saw during total knee arthroplasty: a randomised clinical trial. Knee, 2017, 24: 1213–1220. 28823809 10.1016/j.knee.2017.07.018

[15] Plaskos C , Hodgson AJ , Inkpen K , McGraw RW . Bone cutting errors in total knee arthroplasty. J Arthroplasty, 2002, 17: 698–705. 12216022 10.1054/arth.2002.33564

[16] Lizaur‐Utrilla A , Miralles‐Munoz FA , Sanz‐Reig J , Collados‐Maestre I . Cementless total knee arthroplasty in obese patients: a prospective matched study with follow‐up of 5‐10 years. J Arthroplasty, 2014, 29: 1192–1196. 24355257 10.1016/j.arth.2013.11.011

[17] Sorrells RB , Murphy JA , Sheridan KC , Wasielewski RC . The effect of varus and valgus deformity on results of cementless mobile bearing TKA. Knee, 2007, 14: 284–288. 17561401 10.1016/j.knee.2007.04.004

[18] Paley D . Principles of deformity correction. Berlin Heidelberg: Springer‐Verlag, 2002; 8–10.

[19] Patil S , D'Lima DD , Fait JM , Colwell CW Jr . Improving tibial component coronal alignment during total knee arthroplasty with use of a tibial planing device. J Bone Joint Surg Am, 2007, 89: 381–387. 17272454 10.2106/JBJS.F.00204

[20] Toksvig‐Larsen S , Ryd L . Surface flatness after bone cutting. A cadaver study of tibial condyles. Acta Orthop Scand, 1991, 62: 15–18. 2003380 10.3109/17453679108993084

[21] Macdonald W , Styf J , Carlsson LV , Jacobsson CM . Improved tibial cutting accuracy in knee arthroplasty. Med Eng Phys, 2004, 26: 807–812. 15564118 10.1016/j.medengphy.2004.06.006

[22] Wu PH , Zhang ZQ , Fang SY , *et al* Preoperative measurement of tibial resection in total knee arthroplasty improves accuracy of postoperative limb alignment restoration. Chin Med J (Engl), 2016, 129: 2524–2529. 27779156 10.4103/0366-6999.192789 PMC5125328

[23] Mannan A , Vun J , Lodge C , Eyre‐Brook A , Jones S . Increased precision of coronal plane outcomes in robotic‐assisted total knee arthroplasty: a systematic review and meta‐analysis. Surgeon, 2018, 16: 237–244. 29439922 10.1016/j.surge.2017.12.003

[24] Hetaimish BM , Khan MM , Simunovic N , Al‐Harbi HH , Bhandari M , Zalzal PK . Meta‐analysis of navigation vs conventional total knee arthroplasty. J Arthroplasty, 2012, 27: 1177–1182. 22333865 10.1016/j.arth.2011.12.028

[25] Salzmann M , Fennema P , Becker R , Hommel H . Does postoperative mechanical axis alignment have an effect on clinical outcome of primary total knee arthroplasty? a retrospective cohort study. Open Orthop J, 2017, 11: 1330–1336. 29290871 10.2174/1874325001711011330 PMC5721318

[26] Tsukeoka T , Tsuneizumi Y . Residual medial tightness in extension is corrected spontaneously after total knee arthroplasty in varus knees. Knee Surg Sports Traumatol Arthrosc, 2018, 27: 692–697. 29728741 10.1007/s00167-018-4967-6

[27] Mine T , Hoshi K , Gamada K , *et al* Kinematic analysis of posterior‐stabilized total knee arthroplasty during standing up from and sitting down on a chair. J Orthop Surg Res, 2016, 11: 142. 27855716 10.1186/s13018-016-0482-y PMC5114786

